# Idiopathic Intracranial Hypertension in the United States: Demographic and Socioeconomic Disparities

**DOI:** 10.3389/fneur.2020.00869

**Published:** 2020-09-08

**Authors:** Arash Ghaffari-Rafi, Rana Mehdizadeh, Andrew Wai Kei Ko, Shadeh Ghaffari-Rafi, Jose Leon-Rojas

**Affiliations:** ^1^John A. Burns School of Medicine, University of Hawai'i at Mānoa, Honolulu, HI, United States; ^2^Queen Square Institute of Neurology, University College London, London, United Kingdom; ^3^Faculty of Medicine, University of Queensland, Brisbane, QLD, Australia; ^4^Carver College of Medicine, University of Iowa, Iowa City, IA, United States; ^5^Universidad Internacional del Ecuador Escuela de Medicina, Quito, Ecuador

**Keywords:** idiopathic intracranial hypertension, socioeconomic, demographics, disparities, incidence, United States, income

## Abstract

**Background:** Obesity's risk increases for low-income, female, young, and Black patients. By extrapolation, idiopathic intracranial hypertension (IIH)—a disease associated with body mass index—would potentially display socioeconomic and demographic disparities.

**Methods:** IIH incidence (per 100,000) was investigated with respect to sex, age, income, residence, and race/ethnicity, by querying the largest United States (US) healthcare administrative dataset (1997–2016), the National (Nationwide) Inpatient Sample.

**Results:** Annual national incidence (with 25th and 75th quartiles) for IIH was 1.15 (0.91, 1.44). Females had an incidence of 1.97 (1.48, 2.48), larger (*p* = 0.0000038) than males at 0.36 (0.26, 0.38). Regarding age, largest incidence was among those 18–44 years old at 2.47 (1.84, 2.73). Low-income patients had an incidence of 1.56 (1.47, 1.82), larger (*p* = 0.00024) than the 1.21 (1.01, 1.36) of the middle/high. No differences (χ^2^ = 4.67, *p* = 0.097) were appreciated between urban (1.44; 1.40, 1.61), suburban (1.30; 1.09, 1.40), or rural (1.46; 1.40, 1.48) communities. For race/ethnicity (χ^2^ = 57, *p* = 2.57 × 10^−12^), incidence was largest for Blacks (2.05; 1.76, 2.74), followed by Whites (1.04; 0.79, 1.41), Hispanics (0.67; 0.57, 0.94), and Asian/Pacific Islanders (0.16; 0.11, 0.19). Year-to-year, incidence rose for all strata subsets except Asian/Pacific Islanders (τ = −0.84, *p* = 0.00000068).

**Conclusion:** IIH demonstrates several sociodemographic disparities. Specifically, incidences are larger for those low-income, Black, 18–44 years old, or female, while annually increasing for all subsets, except Asian/Pacific Islanders. Hence, IIH differentially afflicts the US population, yielding in healthcare inequalities.

## Introduction

Characterized by an elevated intracranial pressure and normal cerebrospinal fluid composition, in the absence of enlarged ventricles or space-occupying lesions, idiopathic intracranial hypertension (IIH) is a syndrome without a recognized etiology (1). Yet what has been recognized is 71–94% of IIH patients are comorbid with obesity (2–5). Itself, obesity's risk increases for those who are low income, female, young, or Black (6, 7). Therefore, extrapolation would suggest the presence of disparities in IIH diagnoses, when stratifying against the background of socioeconomic and demographic strata.

Notably, there is impetus to better define IIH's distribution among the subsets of society, especially when considering the burden imposed on patients—as 68–98% suffer from headaches, whereas roughly half experience sustained vision loss and 8–10% bi- or monocular blindness (2, 4, 8–12). Moreover, epidemiology not only provides a conduit for risk-factor identification and by extension etiology elucidation but also aids legislators in addressing community needs (13).

The large diverse population of the United States (US) enables the ability to investigate disease with respect to sociodemographic variables. Hence, we used the largest US administrative dataset [i.e., the National (Nationwide) Inpatient Sample (NIS)] to explore IIH incidence (1997–2016) across the strata of sex, age, income status, residence, and race/ethnicity (14, 15).

## Methods

### Design and Setting

Data were collected from NIS, the US's largest all-payor directory of inpatient hospital admissions—constructed by the Agency of Healthcare Research and Quality (Rockville, MD) as part of the Healthcare Cost and Utilization Project (HCUP) (14, 15). Estimates were calculated after collecting a 20% stratified sample of all community hospital discharges (www.hcup-us.ahrq.gov) (14). As a publicly available database without patient or hospital identifiers, institutional review board exemption for waivers of informed consent was attained from the University of Hawai‘i at Mānoa, Office of Research Compliance.

From 1997 to 2016, the NIS was queried using the International Classification of Diseases 9th or 10th edition Clinical Modification (ICD-9-CM or ICD-10-CM) codes for idiopathic intracranial hypertension: ICD-9-CM (348.2) for 1997–2014; ICD-10-CM (G93.2) for 2015–2016. Then for investigating the role of socioeconomic and demographic variables on IIH, the NIS was probed for the following patient characteristics: sex, age, income, location of residence, and race/ethnicity.

Age was subdivided as 1–17, 18–44, and 45–64 years old (14). Patient income was classified as low or middle/high; HCUP determined income via the surrogate of median household income from the Zone Improvement Plan code of the patient's residence (14). Low income was defined by the first quartile of median household income in the US (from the respective year), whereas middle/high combined all other quartiles (14). Patient's residence was categorized as urban (large central metro), suburban (large fringe metro, or medium and small metro), or rural (micropolitan and non-core) (14). Lastly, race/ethnicity was stratified as White, Black, Hispanic, or Asian/Pacific Islander (14). For tabulating number of IIH cases, weights provided by HCUP were applied, with national numbers confirmed on HCUPnet (https://hcupnet.ahrq.gov).

### Statistical Analysis

For individual subsets of socioeconomic and demographic variables, IIH incidences per 100,000 people were calculated. After acquiring stratified population data from the US Census Bureau (http://www.census.gov), case numbers were normalized to the corresponding US population value for each characteristic (sex, age, income, residence, race/ethnicity); for instance, the number of Blacks diagnosed with IIH in 2010 was divided by the 2010 US Black population. Incidence was presented as the annual median with the interquartile range (25th quartile to 75th quartile) for 1997–2016, unless otherwise stated. Parametric assumptions were not met, hence non-parametric tests were used. For determining incidence trends over the years, the Mann–Kendall test was applied (16, 17). To compare any with two subsets, the Wilcoxon signed-rank test was applied, whereas for studying differences in annual trends in the strata of age, residence, and race/ethnicity, the Friedman rank sum test was used (18, 19). Tests were all two-sided and set with an alpha level of 0.05 as the threshold for statistical significance. The R Statistical Software (R Foundation for Statistical Computing, Vienna, Austria) was used for all analyses (20).

## Results

### Overall Incidence

Annual median incidence (with 25th and 75th quartiles) for IIH (1997–2016) was 1.15 (0.91 to 1.44) and increasing, with a Kendall's τ of 0.78 (*p* = 0.0000038) (Table 1).

**Table 1:** Median annual incidence of idiopathic intracranial hypertension across socioeconomic and demographic factors.

	**Median (25% quartile, 75% quartile) incidence per 100,000**	**Mann–Kendall test**	**Friedman rank sum test**	**Wilcoxon signed rank test (estimated difference between groups)**		
**Overall incidence 1997–2016**	1.15 (0.91, 1.44)	τ= 0.78, *p* = 0.0000038				
**Sex (1997–2016)**						
Female	1.97 (1.48, 2.48)	τ= 0.88, *p* = 0.00000012		1.67 (95% CI: 1.41 to 1.92) *V* = 190, *p* = 0.0000038		
Male	0.36 (0.26, 0.38)	τ= 0.52, *p* = 0.0021				
**Age (1999–2016)**						
1–17 Years	0.89 (0.78, 1.06)	τ= 0.66, *p* = 0.000089	χ^2^= 38 df = 2 *p* = 5.60 × 10^−9^	**Ages 1–17 vs. 18–44**	**Ages 1–17 vs. 45–64**	**Ages 18–44 vs. 45–64**
				1.47 (95% CI: 1.33 to 1.62) *V* = 0, *p* = 0.0000038	0.37 (95% CI: 0.28 to 0.45) *V* = 0, *p* = 0.0000038	1.82 (95% CI: 1.65 to 2.01) *V* = 190, *p* = 0.0000038
18–44 Years	2.47 (1.84, 2.73)	τ= 0.68, *p* = 0.000049				
45–64 Years	0.53 (0.36, 0.63)	τ= 0.73, *p* = 0.000014				
**Patient income (2003–2016)**						
Low income	1.56 (1.47, 1.82)	τ= 0.54, *p* = 0.012		0.44 (95% CI: 0.33 to 0.53) V = 91, *p* = 0.00024		
Middle/high income	1.21 (1.01, 1.36)	τ= 0.72, *p* = 0.00079				
**Patient residence (2007–2016)**						
Large central metro (urban)	1.44 (1.40, 1.61)	τ= 0.56, *p* = 0.048	χ^2^= 4.67 df = 2 *p* = 0.097	**Urban vs. Suburban**	**Urban vs. Rural**	**Suburban vs. Rural**
				0.19 (95% CI: −0.096 to 0.50) *V* = 58, *p* = 0.14	0.018 (95% CI: −0.19 to 0.24) *V* = 26, *p* = 0.73	0.18 (95% CI: −0.017 to 0.33) *V* = 6, *p* = 0.055
Suburban	1.30 (1.09, 1.40)	τ = 0.83, *p* = 0.0025				
Micropolitan and Non-core (Rural)	1.46 (1.40, 1.48)	τ= 0.67, *p* = 0.016				
**Race/Ethnicity (1997–2016)**						
Black	2.05 (1.76, 2.74)	τ= 0.81, *p* = 0.0000014	χ^2^= 57 df = 3 *p* = 2.57 × 10^−12^	**White vs. Black**	**White vs. Hispanic**	**White vs. Asian/Pacific Islander**
				1.14 (95% CI: 0.97 to 1.29) *V* = 0, *p* = 0.0000038	0.35 (95% CI: 0.27 to 0.44) *V* = 190, *p* = 0.0000038	0.91 (95% CI: 0.68 to 1.13) *V* = 190, *p* = 0.0000038
White	1.04 (0.79, 1.41)	τ= 0.81, *p* = 0.0000014				
Hispanic	0.67 (0.57, 0.94)	τ= 0.70, *p* = 0.000037		**Black vs. Hispanic**	**Black vs. Asian/Pacific Islander**	**Hispanic vs. Asian/Pacific Islander**
				1.47 (95% CI: 1.28 to 1.73) *V* = 190, *p* = 0.0000038	2.05 (95% CI: 1.76 to 2.37) *V* = 0, *p* = 0.0000038	0.58 (95% CI: 0.43 to 0.73) *V* = 0, *p* = 0.0000038
Asian/Pacific Islander	0.16 (0.11, 0.19)	τ= −0.84, *p* = 0.00000068				

### Sex

**Figure 1:**
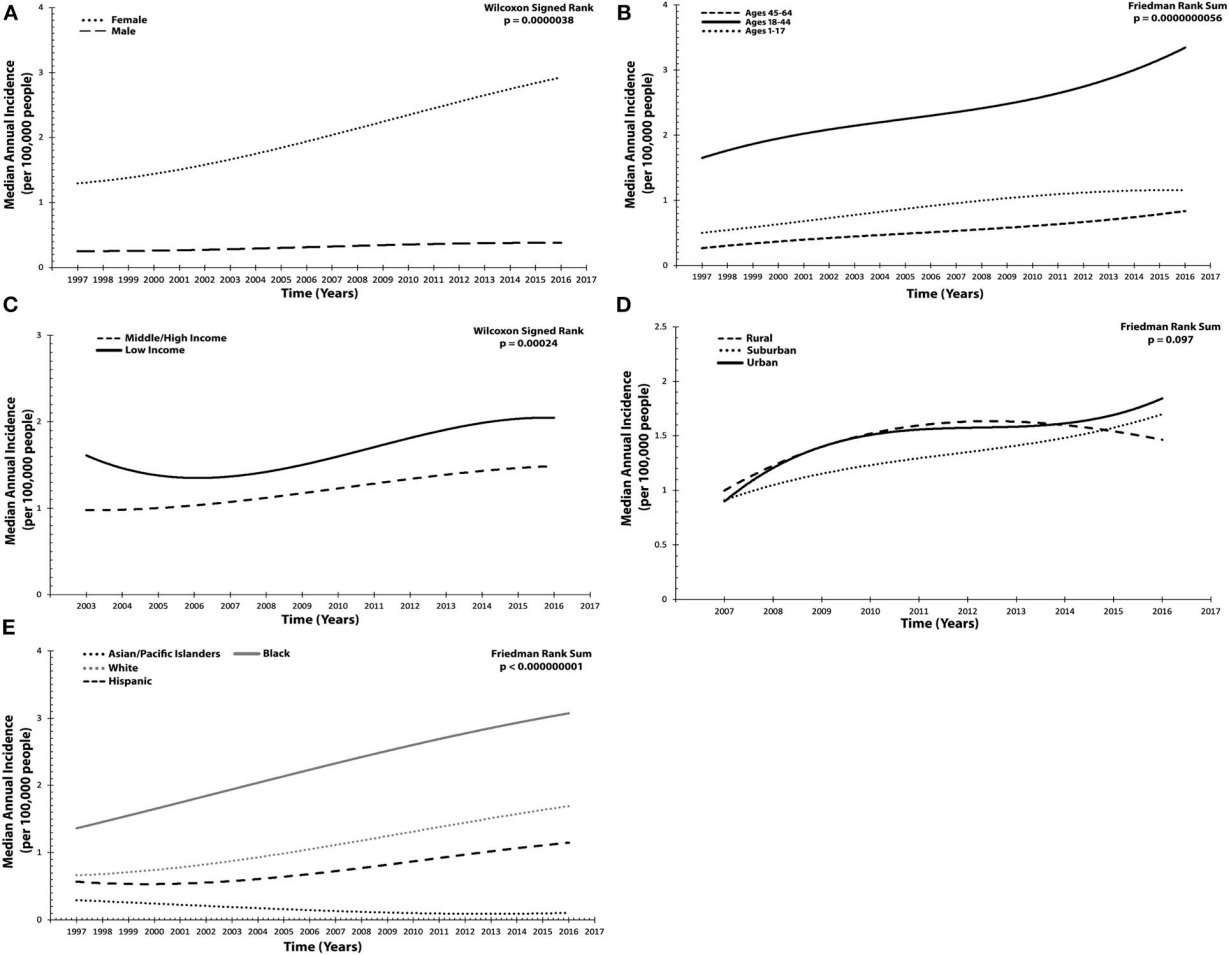
Annual incidence trended for idiopathic intracranial hypertension across socioeconomic and demographic variables per 100,000 people. **(A)** Sex: females, males. **(B)** Age: 1–17 years old, 18–44, and 45–64. **(C)** Patient income status: middle/high income, low income. **(D)** Location of patient residence: urban, suburban, rural. **(E)** Race/Ethnicity: White, Black, Hispanic, Asian/Pacific Islander. Results presented in **(A–E)** were graphed utilized a polynomial model.

From 1997 to 2016, median incidence among females in the US was 1.97 (1.48 to 2.48), which was annually increasing (τ = 0.88, *p* = 0.00000012). Male incidence was 0.36 (0.26 to 0.38) and likewise rising (τ = 0.52, *p* = 0.0021). Compared, female incidence was statistically larger, with an estimated difference of 1.67 (95% CI 1.41 to 1.92, *p* = 0.0000038) (Table 1 and Figure 1A).

### Age

Age was stratified into three groups and assessed from 1999 to 2016. Patients 1–17 years old had an incidence of 0.89 (0.78 to 1.06), which was annually increasing (τ = 0.66, *p* = 0.000089). For those 18–44 years old, incidence was 2.47 (1.84 to 2.73) and likewise significantly increasing (τ = 0.68, *p* = 0.000049). Finally, in the oldest age group, 45–64 years old, median annual incidence was 0.53 (0.36 to 0.63) and also increasing (τ = 0.73, *p* = 0.000014). Statistically significant differences were found between all strata (χ^2^ = 38, *p* = 5.60 × 10^−9^) when compared with one another. Of note, the difference in incidence was largest (1.82, 95% CI 1.65 to 2.01, *p* = 0.0000038) between those 18–44 and 45–64 (Table 1 and Figure 1B).

### Patient Income

Between 2003 and 2016, patient income status was examined. Those from the lowest income quartile had a median incidence of 1.56 (1.47 to 1.82), which was annually increasing (τ = 0.54, *p* = 0.012). For middle/high-income patients, incidence was 1.21 (1.01 to 1.36), likewise increasing (τ = 0.72, *p* = 0.00079). When compared, patients in the low-income subgroup had a significantly larger incidence (0.44, 95% CI 0.33 to 0.53, *p* = 0.00024) (Table 1 and Figure 1C).

### Patient Residence

Subsequently, the location of patient residence (rural, suburban, or urban) was also investigated (2007–2016). Rural communities exhibited an annual incidence of 1.46 (1.40 to 1.48), which was increasing (τ = 0.67, *p* = 0.016). Urban centers had an incidence of 1.44 (1.40 to 1.61), also annually increasing (τ = 0.56, *p* = 0.048). Finally, in suburban communities, incidence was 1.30 (1.09 to 1.40) and increasing (τ = 0.83, *p* = 0.0025). Upon comparing all three geographic regions, no statistically significant differences were found (χ^2^ = 4.67, *p* = 0.097) (Table 1 and Figure 1D).

### Race/Ethnicity

To discern whether disparities in incidence by race/ethnicity exist, four subgroups were examined from 1997 to 2016: Black, White, Hispanic, and Asian/Pacific Islander. Incidence for Blacks was 2.05 (1.76 to 2.74) and annually increasing (τ = 0.81, *p* = 0.0000014). For Whites, incidence was 1.04 (0.79 to 1.41), likewise increasing (τ = 0.81, *p* = 0.0000014). Among Hispanics, incidence was 0.67 (0.57 to 0.94), also with a positive annual trend (τ = 0.70, *p* = 0.000037). Asian/Pacific Islanders exhibited an incidence of 0.16 (0.11 to 0.19), with a negative annual trend (τ = −0.84, *p* = 0.00000064). Overall, comparison of any two subgroups resulted in statistically significant differences (χ^2^ = 57, *p* = 2.57 × 10^−12^) (Table 1 and Figure 1E).

## Discussion

### Epidemiology of Idiopathic Intracranial Hypertension—General Considerations

Between 1997 and 2016, national annual incidence in the US (per 100,000 people) was estimated at 1.15 (0.91 to 1.44) and found to be significantly increasing (τ = 0.78, *p* = 0.0000038). Although no previous nationwide US investigations were available for comparison, there was compatibility of our results with a retrospective population-based cohort study of Olmsted County (Minnesota), which identified an incidence of 1.8 (95% CI 1.3 to 2.2) between 1990 and 2014; similarly, incidence was demonstrated to be increasing from 1990–2001 to 2002–2014 (21). The only other American study (a survey of neurologists, ophthalmologists, and neurosurgeons) was conducted between August 1984 and July 1985, which estimated incidence at 0.9 in Iowa and 1.07 in Louisiana (22).

Internationally, incidence was contingent upon location. In England, a national database demonstrated incidence to be 2.26 in 2002, but then 4.69 in 2016—rising by 108% (23). On the other hand, retrospective chart reviews tabulated incidences of 1.56 (2007–2008) in Sheffield (England) and 0.5 (1991–1995) in Northern Ireland, whereas a prospective study from Fife (Scotland) estimated incidence at 3.56 (2013–2014) (24–26). In Valladolid (Spain) and Parma (Italy), retrospective chart reviews of regional hospitals predicted incidence at to be 3.2 (1994–2004) and 0.28 (1990–1999), respectively (27, 28). Meanwhile, in Benghazi (Libya), Ash Sharqiyah (Oman), and Israel, the respective incidences were 2.2 (1982–1989), 2.18 (2001–2011), and 2.02 (2005–2007) (2, 29, 30). Smallest incidence was in Hokkaido (Japan) at 0.03 (1993) (31).

Although no causative relationship has been established, a robust correlation exists between obesity and IIH incidence, where across cohort studies globally 57–100% of IIH patients were obese (22, 26–30, 32, 33). Of these nations, Japan, which has the smallest IIH incidence, likewise has the lowest obesity prevalence (31, 34). Meanwhile, rising annual incidence in England correlated with increasing body mass index (BMI) (23). Paralleling trends in British data, the increasing annual incidence in our investigation (τ = 0.78, *p* = 0.0000038) correspondingly shadowed the increasing prevalence of obesity in the US (1999–2014) (35). Hence, varying geographic obesity prevalence may participate in yielding differences among international incidences.

Similarly, variations in healthcare systems and accessibility may also contribute to incidence differences. Notably, the larger incidence of 2.2 (1982–1989) in Libya and 4.69 (2016) in England, relative to 1.15 (1997–2016) in the US, could in part arise secondary nearly all Libyan and UK citizens being insured—while 40 million Americans were uninsured in 2003 and 27.5 million in 2017 (8.5% of population) (36–41). Likewise, differing proportions of at-risk demographics (i.e., obese, female, low income, etc.) across cities or nations may also predispose incidences to contrast (34). Yet, notwithstanding these hypotheses, attempts to compare incidences worldwide should be headed with caution, owing to inconsistencies in investigation methodologies and years assessed.

### Sex

In the US (1997–2016), the female-to-male incidence ratio was 5.47, with a median annual incidence of 1.97 (1.48 to 2.48) for females and 0.36 (0.26 to 0.38) for males. Complementing the overall cumulative trend, annual incidences were significantly increasing for both males (τ = 0.52, *p* = 0.0021) and females (τ = 0.88, *p* = 0.00000012). Despite no other nationwide studies for comparison, our results supported observations from regional investigations (21, 22). In the White-predominant Olmsted County (Minnesota), the ratio was 11.3 (1990–2014), with female incidence at 3.277 (95% CI 2.432 to 4.121) and male at 0.290 (95% CI 0.035 to 0.544) (21). Moreover, 1984–1985 data from Iowa and Louisiana determined respective female-to-male ratios of 8.0 and 4.3, with Iowa incidence for 15–44-year-old females at 3.5 and 0.3 for males (22). Relative to our national results, the larger female incidence estimates from Iowa and Minnesota could be secondary to both states having a larger proportion of overweight females than the national median; meanwhile, the similar values for male incidence may be contingent upon the association between BMI and IIH being weaker for males (23, 42).

Elsewhere, in England, incidence was both annually increasing and larger than in the US: female incidence was 3.53 in 2002 and 7.69 in 2016; male incidence was 0.9 in 2002 and 1.6 in 2016 (23). Although our data's female-to-male incidence ratio (5.47) was >3.92 (2002) and 4.81 (2016) from England, we likewise observed the ratio increasing from 1997 to 2016 (23). Regionally, despite a smaller incidence (2007–2008) in Sheffield (England), with females at 2.86 and males at 0.2, the incidence ratio was larger (14.3) (24). Northern Ireland data projected a ratio of 5.73 (1991–1995), with female incidence at 0.86 and male at 0.15 (26). Meanwhile, Benghazi (Libya) data estimated the ratio at 16 (1982–1989), with 4.32 (95% CI 3.40 to 5.40) for female incidence and 0.27 (95% CI 0.00 to 0.63) for male (2). In Ash Sharqiyah (Oman), a female-to-male ratio of 3 (2001–2011) was tabulated, with female incidence at 3.25 (male incidence was not available) (29). A similar female incidence was also estimated in Israel (2005–2007) at 3.17 (95% CI 2.83 to 3.50), with males at 0.85 (95% CI 0.67 to 1.02) and the incidence ratio at 3.73 (30).

Granting that variations in regional obesity prevalence and study methodologies accounted for the different female and male incidences, internationally there remained consistency in females having a greater incidence (2, 21, 22, 26, 29, 30, 34). However, the female predilection does not occur until after puberty, thus advocating a role for sex hormones in pathogenesis (43). One enzyme, 11β-hydroxysteroid dehydrogenase type 1 (11β-HSD1), not only has activity levels which correlate to intracranial pressure but also is differentially modulated by testosterone and exhibits sex differences in regulation (44, 45). Hence, 11β-HSD1—found in both choroid plexus and arachnoid granulation tissue—may play an important role in accounting for the IIH sex differences, as well as IIH's association with BMI (45, 46).

### Age

From the three age strata investigated, between any two categories incidences were statistically different, as well as all annually increasing (1–17 years old: τ = 0.66, *p* = 0.000089; 18–44: τ = 0.68, *p* = 0.000049; 45–64: τ = 0.73, *p* = 0.000014). Peak incidence was among patients 18–44 years old at 2.47 (1.84 to 2.73), whereas the lowest was among those 45–64 years old, at 0.53 (0.36 to 0.63). The youngest age group, 1–17 years old, had an intermediate incidence at 0.89 (0.78 to 1.06). Two previous US investigations on age were conducted, but only one stratified incidence by multiple age categories, albeit different ranges were used relative to our study (21). In Olmsted County (1990–2014), incidence was greatest among those 25–34 years old at 6.084, followed by patients 15–24 at 4.718 (21). Among the youngest age group (0–14 years old), incidence was 0.983, while for the oldest (45+) was 0.181 (21). The absence of Asian/Pacific Islanders and Hispanics—racial/ethnic groups with the smallest incidences in our dataset—likely contributed to the larger peak incidence in Olmsted County (21). Meanwhile, in central Ohio (2010–2013), a study on pediatric (0–18 years old) patients found an incidence of 0.63 (47).

Globally, similar trends in age were observed, with variations in values potentially secondary to differences in population demographics and study methods. In Northern Ireland (1991–1995), peak incidence was among patients 15–24 years old (0.96), followed by 24–35 (0.93); incidence among pediatric patients (0–14) was 0.10, while in the oldest strata (45+) was 0.26 (26). Data from Benghazi (Libya) found peak incidence to be among patients 30–39 years old (9.33), followed by 20–29 (6.37); for pediatric patients (0–14) and the oldest strata (50–59), incidences were 0.11 and 0.62, respectively (2). Finally, in Israel (2005–2007) incidence among patients 17 and younger was found to be 1.75 (95% CI 1.44 to 2.05), while for those older than 17 was 2.07 (95% CI 1.84 to 2.31) (30).

Despite our dataset precluded the ability to sub-stratify age by sex, other investigations have demonstrated the peak incidence (ages 18–44) is secondary to a spike in female diagnoses (23, 30). Hence, based on age, the predilection for IIH occurs not only between puberty and menopause but also parallels the increasing prevalence of obesity in developed nations (overweight/obesity prevalence steadily rises to a peak at 55–60 years old) (34, 48). These findings that peak incidence is during the reproductive years and ages of greatest BMI further lend support to sex hormones and 11β-HSD1 engaging in IIH pathogenesis (43–46).

### Patient Income

Disease morbidity and mortality has long been linked to socioeconomic status, yet no investigations have examined such an association with IIH incidence in the US (13, 49, 50). Between 2003 and 2016, patients in the lowest income quartile were found to have an incidence of 1.56 (1.47 to 1.82), significantly larger (0.44, 95% CI 0.33 to 0.53, *p* = 0.00024) than the 1.21 (1.01 to 1.36) of middle/high-income patients. Of note, the incidence projections for low-income patients are potentially underestimated, as low-income citizens are most likely uninsured, as well as considering 40 million in 2003 and 27.5 million in 2017 of Americans were uninsured (36, 40). Nonetheless, these observations corroborate findings from another study on social determinants of health, which found in England (2002–2016) over half the patients with IIH were from the lowest two quintiles (i.e., greatest social deprivation) on the English index of multiple deprivation—there were also the two quintiles with the greatest obesity rates (23). Upon further stratification, the positive association between IIH and social deprivation was sustained only for females (*r* = 0.89, *p* < 0.001; males: *r* = 0.37, *p* = 0.539), and only among females was deprivation quintile associated with BMI (*r* = 0.98, *p* = 0.003; males: *r* = 0.59, *p* = 0.291) (23). In the US (2011–2014), similar associations are observed; among males, obesity prevalence exhibits no difference between the lowest (31.5%) and highest (32.6%) income strata, yet for females, obesity prevalence increases (29.7 to 45.2%) with falling income (51). Hence, the greater BMI, among low-income female patients, may be one variable which contributes to the socioeconomic disparity in IIH incidence. To better parse the role of BMI in poverty, future investigations should examine IIH incidence within each income strata, but with patients who have a uniform BMI.

### Location of Patient Residence

When examining the distribution of IIH on the basis of geography (urban, suburban, and rural), the year-to-year incidence was significantly increasing in all three subgroups, corresponding to the overall incidence's trend. Itself, incidence was found to be largest in rural communities (1.46), followed by urban (1.44) and suburban (1.30), yet these values were not statistically different (χ^2^ = 4.67, *p* = 0.097). As BMI is theorized to contribute to IIH pathogenesis, the larger incidence in rural communities could be secondary to a greater prevalence of obesity in these regions (52, 53). Indeed, the US Centers for Disease Control and Prevention, 2016 Behavioral Risk Factor Surveillance System, did identify a larger (*p* < 0.001) prevalence of obesity in non-metropolitan (i.e., rural) counties (34.2%) relative to metropolitan (28.7%) (54). However, the lack of statistically significant differences in IIH incidences implies BMI may not play as strong of a role in IIH development. Although no other investigations on patient residence and incidence were available for comparison, our results suggest there is reduced probability of a location-dependent disparity in healthcare access for IIH.

### Race/Ethnicity

Several disparities were identified when examining incidence along the lines of race/ethnicity. Blacks had the largest incidence at 2.05 (1.76 to 2.74), followed by Whites at 1.04 (0.79 to 1.41), Hispanics at 0.67 (0.57 to 0.94), and Asian/Pacific Islanders at 0.16 (0.11 to 0.19); these values were all statistically different (χ^2^ = 57, *p* = 2.57 × 10^−12^). When investigating year-to-year trends, for Blacks (τ = 0.81, *p* = 0.0000014), Whites (τ = 0.81, *p* = 0.0000014), and Hispanics (τ = 0.70, *p* = 0.000037) incidences were annually increasing, yet for Asian/Pacific Islanders (τ = −0.84, *p* = 0.00000068) incidence was decreasing. No previous investigations had examined incidence with regards to race/ethnicity or identified racial/ethnic disparities in IIH.

The large incidence among Blacks may be secondary to this subgroup having the greatest prevalence of obesity in the US (51, 54). Corroborating the role of BMI, Asian/Pacific Islanders who have the lowest incidence of IIH also have the lowest prevalence of obesity in the US; furthermore, albeit a single datapoint, worldwide the lowest incidence was from Asia [0.03 in Hokkaido, Japan (1993)] (31, 51). Decreasing annual obesity prevalence (1998–2003) among Asians, with rising prevalence for Blacks, Whites, and Hispanics, would also help describe the annual trends in IIH incidence (55). However, if obesity solely dictated IIH incidence, then Hispanics, who have the second largest obesity prevalence, would be expected to have the second largest IIH incidence—which was not the case (54). Although there may be a genetic etiology accounting for the racial/ethnicity disparities in IIH incidence, currently no loci have been found via genome-wide association studies (56).

On the other hand, social determinants of health could contribute to these disparities. For instance, of all subgroups in the US, Hispanics face the most barriers to health insurance access, which in turn may account for the lower IIH incidence, despite the larger obesity prevalence (54, 57). Unrecognized occupational or environmental exposures may also play a role. Blacks are more likely to work in transportation, material moving, production, and service careers, whereas Asian/Pacific Islanders in professional and management occupations associated with greater economic status (58). In relation, Blacks have the lowest median household income, whereas Asian/Pacific Islander have the highest (59). Moreover, as low-income patients have a larger IIH incidence (0.44, 95% CI 0.33 to 0.53, *p* = 0.00024), such implies poverty as a potential confounding variable. Even with poverty controlled, some diseases still exhibit predisposition toward Blacks, thus suggesting a role for other psychosocial stressors, including discrimination—which is experienced greatest among Blacks (60). Discrimination's influence on health disparities is hypothesized to yield from shaping psychological (i.e., anger, depression, etc.) or physiological (i.e., immune, autonomic nervous system, etc.) responses to modulate downstream diseases states (i.e., cardiovascular, cancer, etc.) (60–62). Overall, although the etiology remains unknown, what is known is that IIH incidence exhibits significant racial disparities.

### Limitations

Notwithstanding these results, there are limitations in this retrospective study. As the dataset was of inpatient discharges, such excludes the small fraction of patients incidentally diagnosed or not requiring hospitalization. Also unknown is whether IIH was the primary reason for hospitalization or if the disease was a secondary diagnosis in the patient's records. Moreover, using a national database prevents the ability to confirm whether participating hospitals applied a standardized diagnostic criterion, whereas using ICD-CM codes creates susceptibility to administrative data input errors. Likewise, currently there are no previous investigations validating accuracy for the IIH ICD codes; however, by using a large sample size (i.e., nationwide database) such reduces the influence coding errors at any individual hospital have. In addition, some of the relationships observed may have yielded from confounding (i.e., race/ethnicity incidences may have been influenced by socioeconomic status), hence future investigations should use multivariate analyses if amenable. Lastly, over time there is potentially greater awareness of IIH, meaning detection bias could influence number of diagnoses. Hence, in summary, these results should be interpreted carefully and only in the context of the limitations.

## Conclusion

In summary, IIH was found to exhibit several uncharacterized trends in incidence (per 100,000 people). National incidence (1997–2016) in the US was 1.15 (0.91 to 1.44) and annually increasing. Likewise, incidence was significantly increasing among all sexes, age strata, income categories, and geographic divisions. Disproportionately, those affected were females, ages 18–44, and low income; no differences were found between urban, suburban, and rural communities. Regarding race/ethnicity, Blacks presented with the largest incidence, followed by Whites, Hispanics, and Asian/Pacific Islanders. Moreover, Asian/Pacific Islanders were the only population subset where IIH incidence was decreasing, rather than annually increasing. These results not only provide the first national incidence statistics for the US on IIH but also identify presence of healthcare disparities necessitating intervention.

## Data Availability Statement

The raw data supporting the conclusions of this article will be made available by the authors, without undue reservation.

## Author Contributions

AG-R, RM, AK, SG-R, and JL-R contributed equally to the development, data collection, analysis, and writing of this manuscript. All authors contributed to the article and approved the submitted version.

## Conflict of Interest

The authors declare that the research was conducted in the absence of any commercial or financial relationships that could be construed as a potential conflict of interest.

## Abbreviations

IIH: idiopathic intracranial hypertension
US: United States
NIS: National (Nationwide) Inpatient Sample
HCUP: Healthcare Cost and Utilization Project
ICD-9-CM, ICD-10-CM: International Classification of Diseases 9th or 10th edition Clinical Modification
BMI: body mass index
CI: confidence interval.
